# The interplay of complex PTSD, intimate partner violence, and metacognitive beliefs in driving methamphetamine craving among women: A structural equation modeling study

**DOI:** 10.1016/j.abrep.2026.100699

**Published:** 2026-05-08

**Authors:** Roya Forootan, Shahram Mohammadkhani, Mehdi Akbari, Mark D. Griffiths

**Affiliations:** a Department of Clinical Psychology, Faculty of Psychology and Education, Kharazmi University, Tehran, Iran; b Now retired. Formerly at Nottingham Trent University, Nottingham, UK

**Keywords:** Complex post-traumatic stress disorder, Intimate partner violence, Craving, Metacognitive beliefs, Perceived social support, Methamphetamine use disorder

## Abstract

Craving is a core clinical feature associated with relapse and poorer treatment outcomes in substance use disorders (SUDs), particularly among women. The present study examined psychological and interpersonal factors contributing to craving among women diagnosed with methamphetamine use disorder, with a specific focus on complex post-traumatic stress disorder (CPTSD) and intimate partner violence (IPV). Using structural equation modeling, the study tested whether CPTSD and IPV were directly associated with craving and whether perceived social support and metacognitive beliefs about desire thinking mediated these relationships. Data were collected from 244 women diagnosed with methamphetamine use disorder (M_age = 32.55 years, SD = 8.72) recruited from addiction treatment centers in Tehran and Karaj, Iran. The proposed model demonstrated excellent fit (χ^2^ = 15.75, df = 14, χ^2^/df = 1.12, CFI = 0.997, TLI = 0.985, RMSEA = 0.023, SRMR = 0.031). Both CPTSD and IPV showed significant direct associations with craving. In addition, positive and negative metacognitive beliefs about desire thinking significantly mediated these relationships, whereas perceived social support did not. Overall, the findings emphasize the role of trauma-related psychopathology and maladaptive metacognitive processes in craving and point to key targets for trauma-informed and metacognitive-focused interventions among women with substance use disorders.

## Highlights

• Craving predictors examined in women with methamphetamine use disorder.
• CPTSD and intimate partner violence directly predicted craving.
• Desire thinking metacognitions mediated trauma–craving links.
• Perceived social support showed no mediating effects.
• Results support trauma-informed, metacognitive interventions.

## 1 Introduction

The World Drug Report estimates that approximately 292 million individuals worldwide use illicit substances, with women accounting for a substantial proportion of users of amphetamine-type stimulants (United Nations Office on Drugs and Crime., 2023). Despite evidence that women progress more rapidly from initial substance use to problematic patterns and face distinct barriers to treatment access, particularly for stimulants, addiction research has historically focused on male samples (Back et al., 2011, Mayo et al., 2019). This gender imbalance highlights the need for studies that specifically examine mechanisms underlying substance use–related outcomes among women.

Craving represents a central clinical process implicated in relapse across substance use disorders and is consistently associated with poorer treatment outcomes (Sinha, 2013, Witkiewitz and Marlatt, 2004). Compared to men, women with SUDs often report more intense craving, heightened cue reactivity, and stronger stress-induced craving responses (Beckham et al., 2007, Fox et al., 2005, Kennedy et al., 2013, Moran et al., 2018). Identifying psychological and interpersonal factors that amplify craving among women is therefore of considerable clinical importance.

Trauma-related psychopathology and interpersonal violence are particularly salient for women. Childhood adversity and trauma exposure are strongly associated with craving in later substance use (Garland et al., 2019). Complex post-traumatic stress disorder (CPTSD), as defined in the ICD-11, is conceptualized as lying on the trauma spectrum (Goodman, 2012), arising following prolonged or repeated interpersonal trauma, and is characterized by affect dysregulation, negative self-concept, and persistent relational disturbances (Cloitre et al., 2013, Cloitre et al., 2014, Cloitre et al., 2018, Herman, 1992a, Herman, 1992b). These features compromise emotion regulation and self-awareness (Briere and Rickards, 2007), which are central mechanisms in stress-related craving and relapse processes (Renaud et al., 2021; Sinha, 2013). Empirical evidence further suggests that women with CPTSD experience greater psychiatric severity and are more likely to engage in heavy substance use compared to men (Giarratano et al., 2020).

Intimate partner violence (IPV) represents one of the most prevalent and chronic forms of interpersonal trauma experienced by women (Cascardi et al., 1992) and is a well-established risk factor for the development and maintenance of CPTSD symptoms (Herman, 1992a, World Health Organization, 2021). IPV may therefore function both as an etiological pathway to CPTSD and as an ongoing interpersonal stressor that independently contributes to craving. Despite this conceptual overlap, few studies have examined CPTSD and IPV simultaneously within a unified model of craving among women with substance use disorders. Addressing this gap may clarify how distinct yet interconnected trauma-related experiences shape craving severity.

### 1.1 Theoretical rationale of craving

Drawing on relapse prevention and stress-based models, craving can be conceptualized as emerging from both tonic and phasic processes (Witkiewitz et al., 2014). Within this framework, CPTSD and IPV can be understood as tonic vulnerability factors that heighten baseline susceptibility to craving by undermining emotion regulation, self-efficacy, and perceptions of interpersonal safety. This perspective provides a theoretical rationale for examining CPTSD and IPV as core predictors of craving among women with methamphetamine use disorder.

### 1.2 Other mediators of craving

In addition to trauma-related factors, cognitive and metacognitive processes play a critical role in the intensification and maintenance of craving. Desire thinking is a voluntary, repetitive cognitive process involving the elaboration of positive images and self-talk related to a desired target (Caselli & Spada, 2015; Mansueto et al., 2019). Previous studies have shown that desire thinking predicts craving and is maintained by specific metacognitive beliefs (Caselli et al., 2013, Caselli et al., 2015, Caselli and Spada, 2010, Caselli and Spada, 2011, Caselli and Spada, 2013, Caselli and Spada, 2015, Spada et al., 2013). Metacognitive beliefs about desire thinking—both positive (e.g., beliefs about its usefulness) and negative (e.g., beliefs about its uncontrollability or harmfulness)—have been shown to amplify craving intensity and persistence (Spada et al., 2015). Recent meta-analytic evidence confirms robust associations between maladaptive metacognitive beliefs, desire thinking, and addictive behaviors (Akbari et al., 2026).

Chronic interpersonal trauma, such as CPTSD and IPV, may foster dysfunctional metacognitive beliefs by increasing self-focused attention, emotional dysregulation, and threat monitoring (Myers & Wells, 2015). Therefore, the present study examined metacognitive beliefs about desire thinking as potential mechanisms linking CPTSD and IPV to craving. In addition to metacognitive beliefs, perceived social support was included as a potential mediator. Research indicates that social support, along with irrational beliefs, plays a significant role in craving among drug users (Bo et al., 2007). This is consistent with evidence that trauma exposure is often associated with diminished or disrupted social resources (Dworkin et al., 2019; Akbari et al., 2026).

To our knowledge, this is the first study to simultaneously examine CPTSD and intimate partner violence within a metacognitive framework of craving among women with methamphetamine use disorder. Based on the aforementioned literature, the aim of the present study was to examine a comprehensive model. More specifically, it was hypothesized that (i) CPTSD and IPV would be associated with craving (H_1_), and (ii) metacognitive beliefs about desire thinking and perceived social support would mediate the relationship between CPTSD, IPV, and craving (H_2_).

## 2 Method

### 2.1 Participants and procedure

The study sample comprised 244 women (Mage = 32.55 years, SD = 8.72; range = 18–60 years) diagnosed with methamphetamine use disorder, all of whom were actively engaged in treatment and cessation efforts at the time of data collection. Participants were recruited using convenience sampling from outpatient and residential addiction treatment centers in Tehran and Karaj, Iran.

Inclusion criteria were: (a) being aged between 18 and 60 years; (b) having a history of methamphetamine use ranging from 1 to 20 years; (c) having current involvement in a romantic relationship to allow assessment of recent intimate partner violence; (d) having basic literacy sufficient to complete self-report questionnaires; (e) having a willingness to participate; and (f) being abstinent from substances for at least 7–10 days prior to assessment to minimize the influence of acute withdrawal symptoms on craving reports.

Exclusion criteria included: (a) presence of intellectual disability or severe psychiatric disorders (e.g., active psychotic disorders); (b) presence of severe physical illness; and (c) unwillingness to participate. Severe psychiatric disorders and intellectual disability were assessed through review of clinical records and routine psychiatric evaluations conducted by treatment center clinicians, based on DSM-5 diagnostic criteria (American Psychiatric Association, 2014). Participants presenting with hallucinations or delusions at intake were excluded. Physical illnesses were assessed through medical records maintained by the treatment centers. The absence of structured diagnostic interviews represents a methodological limitation and is acknowledged as such.

Of the 294 women initially screened, 10 were excluded due to serious physical illnesses and two were excluded due to active psychotic symptoms. The remaining 282 eligible participants received detailed verbal and written information about the study and provided written informed consent. Eight participants subsequently declined participation and 30 were excluded due to incomplete questionnaires, resulting in a final sample of 244 participants.

### 2.2 Ethical Considerations

The study followed all ethical guidelines with the subjects. The procedure was conducted and approved in accordance with ethical standards of the Ethics Committee of Kharazmi University of Tehran (Iran) and adhering to the Declaration of Helsinki. Participants were assured of confidentiality, anonymity, and their right to withdraw from the study at any time without consequences for their treatment.

### 2.3 Measures

#### 2.3.1 Demographic and substance use information

Participants provided demographic information including age, education level, employment status, and perceived financial status. Substance use history was assessed using self-report items addressing duration of methamphetamine use (in years), time since last use (in days), and primary route of administration.

#### 2.3.2 Craving Questionnaire (CQ)

The 20-item Persian version of the Craving Questionnaire (CQ) was used to assess post-cessation substance craving, including thoughts, fantasies, and impulses related to substance use (Tiffany & Drobes, 1991). The Persian version has been used in previous drug attention control training studies (Salehi Fadardi and Bar Erfan, 2010). Items (e.g., *“Thoughts of substances and craving interfere with important tasks”*) are rated on a six-point Likert scale ranging from 0 (*strongly disagree*) to 5 (*strongly agree*). Total scores range from 0 to 100, with higher scores indicating greater craving intensity. The Persian adaptation demonstrated excellent internal consistency in the present sample (Cronbach’s α = 0.83).

#### 2.3.3 International Trauma Questionnaire (ITQ)

Complex post-traumatic stress disorder and PTSD symptoms were assessed using the International Trauma Questionnaire (ITQ) (Cloitre et al., 2018). The ITQ consists of 12 items assessing PTSD symptoms and disturbances in self-organization (DSO). Items are rated on a 5-point Likert scale from 0 (*not at all*) to 4 (*extremely*), referring to experiences during the past month. Higher scores reflect greater trauma-related symptom severity. The Persian version (Benrazi Ghabeshi et al., 2021) showed excellent internal consistency in the present study (Cronbach’s α = 0.926).

#### 2.3.4 Questionnaire for Measuring Violence Against Women (QMVAM)

Intimate partner violence was assessed using the Questionnaire for Measuring Violence Against Women (QMVAM) (Haj-Yahia, 1999, Haj-Yahia, 2000). The scale includes 32 items covering psychological, physical, sexual, and economic violence. Items are rated on a three-point scale from 1 (*never*) to 3 (*twice or more*). Higher total scores indicate greater severity of IPV. Internal consistency in the present study was excellent (Cronbach’s α = 0.957).

#### 2.3.5 Multidimensional Scale of Perceived Social Support (MSPSS)

Perceived social support was assessed using the Multidimensional Scale of Perceived Social Support (MSPSS) (Zimet et al., 1988), consisting of 12 items assessing support from family, friends, and significant others. Items are rated on a seven-point Likert scale from 1 (*strongly disagree*) to 7 (*strongly agree*). The Persian version (Besharat, 2006) demonstrated excellent internal consistency (Cronbach’s α = 0.928).

#### 2.3.6 Metacognitions about Desire Thinking Questionnaire (MDTQ)

Metacognitive beliefs about desire thinking were assessed using the Metacognitions about Desire Thinking Questionnaire (MDTQ) (Caselli & Spada, 2013). The MDTQ includes 18 items assessing positive metacognitions, negative metacognitions, and need to control thoughts. Items are rated on a four-point Likert scale from 1 (*do not agree*) to 4 (*agree very much*). Higher scores indicate stronger maladaptive metacognitive beliefs. The Persian version (Karami et al., 2021) demonstrated very good internal consistency in the present study (Cronbach’s α = 0.894).

### 2.4 Data analysis

Statistical analyses were conducted using *SPSS (Version 27)* and *R (Version 4.4.1)*. A three-step analytic strategy was employed: (i) evaluation of descriptive statistics and internal consistency of measures; (ii) Pearson correlation analyses to examine bivariate associations; and (iii) structural equation modeling (SEM) to test the hypothesized model.

Given evidence of non-normal multivariate distribution (using Mardia’s test), the Weighted Least Squares Mean and Variance adjusted (WLSMV) estimator was used for SEM analyses (Hu & Bentler, 1999). Indirect effects were tested using bootstrapping with 1,000 resamples. Model fit was evaluated using established criteria: CFI (comparative fit index) and TLI (Tucker-Lewis index) ≥ 0.95, RMSEA (root mean square error of approximation) ≤ 0.06, and SRMR (standardized root mean square residual) ≤ 0.08. Demographic variables were included as covariates to control for potential confounding effects.

## 3 Results

### 3.1 Participant Demographic Characteristics

The final sample consisted of 244 women diagnosed with methamphetamine use disorder. Participants were aged between 18 and 60 years, with the majority in the 31–40-year age range (44.7%), followed by 18–30 years (40.6%), 41–50 years (12.3%), and 51–60 years (2.5%). Regarding employment status, 57.8% were unemployed, 20.1% were employed part-time, and 22.1% were employed full-time. In terms of educational attainment, 43.0% had less than a high school diploma, 38.9% had completed high school, 15.2% held a bachelor’s degree, and 2.9% had a master’s degree or higher. Financial status was reported as low (24.6%), average (61.1%), or high (14.3%).

With respect to substance use history, duration of methamphetamine use ranged from 1 to 20 years: 38.5% reported 1–5 years, 32.0% reported 6–10 years, 14.8% reported 11–15 years, and 14.8% reported 16–20 years of use. Abstinence duration at assessment was less than 30 days (48.8%), 31–90 days (30.3%), 91–180 days (15.2%), or 181–365 days (5.7%). The primary route of methamphetamine administration was smoking (98.4%), followed by oral ingestion (1.2%) and injection (0.4%).

### 3.2 Descriptive statistics and correlations

Descriptive statistics (means, standard deviations, minimums, maximums, skewness, and kurtosis) are presented in Table 1, indicating acceptable distributional properties for all variables.

**Table 1:** Descriptive indices of research variables*.*

**Variable**	***M***	***SD***	**Min**	**Max**	**Skewness**	**Kurtosis**
Craving (CQ)	39.74	29.63	0	100	0.17	−1.32
CPTSD symptoms (ITQ)	48.98	22.41	6	88	−0.22	−1.26
Psychological violence (QMVAM)	32.20	10.13	16	48	−0.15	−1.27
Physical violence (QMVAM)	19.72	8.71	11	33	0.37	−1.51
Sexual violence (QMVAM)	4.69	2.35	3	9	0.91	−0.85
Economic violence (QMVAM)	3.33	1.67	2	6	0.68	−1.26
Intimate partner violence (QMVAM)	59.94	19.15	32	96	0.03	−1.34
Positive metacognitions (MDTQ-P)	12.95	6.53	8	32	1.17	0.23
Negative metacognitions (MDTQ-N)	12.60	5.83	6	24	0.57	−0.97
Need to control thoughts (MDTQ-C)	8.70	3.93	4	16	0.65	−0.90
Perceived social support (MSPSS)	53.87	22.49	12	84	−0.30	−1.14

**Note.***M* = mean; *SD* = standard deviation. CQ = Craving Questionnaire; ITQ = International Trauma Questionnaire; QMVAM = Questionnaire for Measuring Violence Against Women; MDTQ = Metacognitions about Desire Thinking Questionnaire; MSPSS = Multidimensional Scale of Perceived Social Support.

Pearson correlation analyses (Table 2) showed significant associations among the study variables. Craving (CQ) was moderately to strongly correlated with CPTSD symptoms (ITQ) (*r* = 0.62, *p* < 0.001), intimate partner violence (QMVAM) (*r* = 0.49, *p* < 0.001), and metacognitive beliefs about desire thinking (MDTQ) (*r* = 0.75, *p* < 0.001). IPV showed moderate positive correlations with CPTSD (*r* = 0.43, *p* < 0.001) and metacognitive beliefs (*r* = 0.44, *p* < 0.001). CPTSD was also moderately correlated with metacognitive beliefs (*r* = 0.60, *p* < 0.001). Perceived social support (MSPSS) showed significant negative correlations with craving (*r* =  − 0.25, *p* < 0.001), CPTSD (*r* =  − 0.27, *p* < 0.001), IPV (*r* =  − 0.28, *p* < 0.001), and metacognitive beliefs (*r* =  − 0.34, *p* < 0.001).

**Table 2:** Pearson correlations among study variables.

	**1**	**2**	**3**	**4**	**5**	**6**	**7**	**8**	**9**	**10**	**11**	**12**	**13**	**14**	**15**
1. Craving (CQ)	1														
2. CPTSD symptoms (ITQ)	0.62***	1													
3. Psychological violence (QMVAM)	0.47***	0.43***	1												
4. Physical violence (QMVAM)	0.38***	0.33***	0.68***	1											
5. Sexual violence (QMVAM)	0.32***	0.26***	0.43***	0.35***	1										
6. Economic violence (QMVAM)	0.30***	0.23***	0.44***	0.29***	0.32***	1									
7. Intimate partner violence (QMVAM)	0.49***	0.43***	0.93***	0.88***	0.54***	0.49***	1								
8. Positive metacognitions (MDTQ-P)	0.57***	0.40***	0.33***	0.27***	0.15*	0.24***	0.34***	1							
9. Negative metacognitions (MDTQ-N)	0.73***	0.60***	0.37***	0.27***	0.29***	0.27***	0.38***	0.54***	1						
10. Need to control thoughts (MDTQ-C)	0.31***	0.31***	0.25***	0.19**	0.20**	0.13*	0.26***	0.05	0.29***	1					
11. Metacognitive beliefs (MDTQ)	0.75***	0.60***	0.44***	0.34***	0.28***	0.30***	0.44***	0.81***	0.86***	0.49***	1				
12. Support from significant others	−0.25***	−0.26***	−0.28***	−0.27***	−0.16*	−0.17**	−0.31***	−0.27***	−0.16*	−0.22***	−0.29***	1			
13. Support from family	−0.27***	−0.30***	−0.28***	−0.27***	−0.19**	−0.15*	−0.31***	−0.22***	−0.25***	−0.24***	−0.31***	0.66***	1		
14. Support from friend	−0.10	−0.12	−0.11	−0.08	0.01	−0.06	−0.10	−0.15*	−0.11	−0.29***	−0.22***	0.46***	0.40***	1	
15. Perceived social support (MSPSS)	−0.25***	−0.27***	−0.27***	−0.25***	−0.13*	−0.15*	−0.28*	−0.26***	−0.21***	−0.31***	−0.34***	0.84***	0.83***	0.78***	1

**Note.***N* = 244. CQ = Craving Questionnaire; ITQ = International Trauma Questionnaire; QMVAM = Questionnaire for Measuring Violence Against Women; MDTQ = Metacognitions about Desire Thinking Questionnaire; MSPSS = Multidimensional Scale of Perceived Social Support.

*p* < 0.05*, *p* < 0.01**, *p* < 0.001***.

### 3.3 Structural equation modeling

**Fig. 1:**
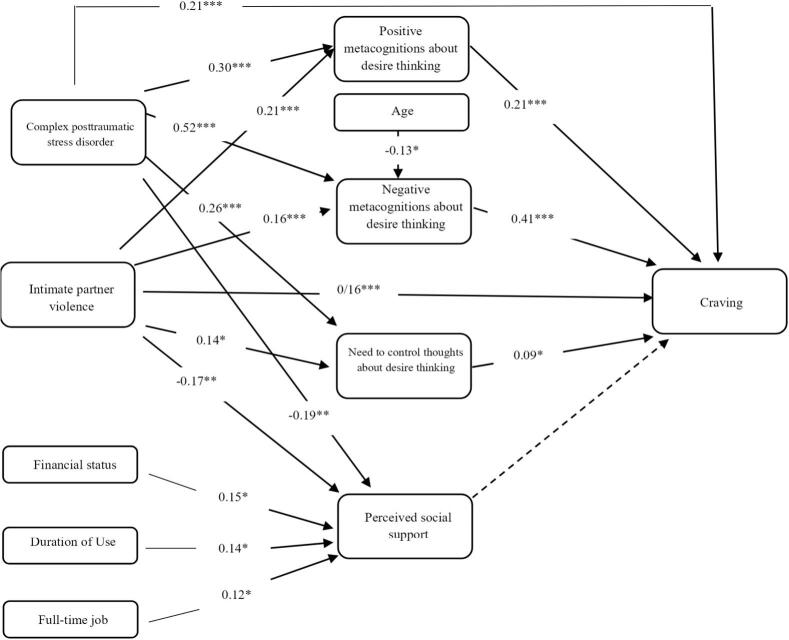
*Standardized Path Coefficients of the Structural Model.***Note.** Standardized coefficients are shown. CPTSD = Complex Post-Traumatic Stress Disorder (ITQ); IPV = Intimate Partner Violence (QMVAM); CQ = Craving Questionnaire; MDTQ = Metacognitions about Desire Thinking Questionnaire. *p* < 0.05*, *p* < 0.01**, *p* < 0.001***.

The measurement model demonstrated good fit (χ^2^ = 9086.46, df = 5186, χ^2^/df = 1.75, CFI = 0.993, TLI = 0.991, RMSEA = 0.056, SRMR = 0.087). The structural model was then evaluated. As shown in Fig. 1, CPTSD and IPV exhibited significant direct effects on craving, supporting H_1_. The model explained a substantial proportion of variance in craving (*R*^2^ = 0.65). Explained variance was moderate for positive metacognitions (*R*^2^ = 0.20), negative metacognitions (R^2^ = 0.38), need to control thoughts (*R*^2^ = 0.13), and perceived social support (*R*^2^ = 0.16).

### 3.4 Mediation analysis

Bootstrapped mediation analyses (Table 3) indicated that positive and negative metacognitive beliefs about desire thinking significantly mediated the associations between both IPV and CPTSD with craving, partially supporting H_2_.

**Table 3:** Standardized indirect effects of CPTSD and intimate partner violence on craving.

**Predictor**	**Mediator**	**β**	**SE**	**95% CI**	**p**
IPV	Positive metacognitions (MDTQ-P)	0.043	0.018	[0.017, 0.117]	0.002
IPV	Negative metacognitions (MDTQ-N)	0.066	0.024	[0.028, 0.176]	0.001
IPV	Need to control thoughts (MDTQ-C)	0.012	0.010	[−0.004, 0.031]	0.118
CPTSD	Positive metacognitions (MDTQ-P)	0.062	0.021	[0.032, 0.132]	0.001
CPTSD	Negative metacognitions (MDTQ-N)	0.212	0.041	[0.187, 0.372]	< 0.001
CPTSD	Need to control thoughts (MDTQ-C)	0.019	0.014	[−0.006, 0.044]	0.094

**Note.** β = standardized indirect effect. Indirect effects were tested using bootstrapping with 1,000 resamples.

IPV = intimate partner violence (QMVAM); CPTSD = complex post-traumatic stress disorder (ITQ); MDTQ-P = positive metacognitions about desire thinking; MDTQ-N = negative metacognitions about desire thinking; MDTQ-C = need to control thoughts.

For IPV, significant indirect effects were observed via positive metacognitions (β = 0.043, 95% CI [0.017, 0.117]) and negative metacognitions (β = 0.066, 95% CI [0.028, 0.176]), whereas the indirect pathway via need to control thoughts was not significant. For CPTSD, significant indirect effects were identified through positive metacognitions (β = 0.062, 95% CI [0.032, 0.132]) and negative metacognitions (β = 0.212, 95% CI [0.187, 0.372]). In contrast, perceived social support did not significantly mediate the relationships between IPV and craving or between CPTSD and craving.

### 3.5 Model fit and control variables

The final structural model demonstrated excellent fit (χ^2^ = 15.75, df = 14, χ^2^/df = 1.12, CFI = 0.997, TLI = 0.985, RMSEA = 0.023, SRMR = 0.031). Demographic covariates were retained in the model. Significant direct paths were observed between financial status, duration of substance use, and full-time employment with perceived social support, and between age and negative metacognitive beliefs, indicating appropriate control of confounding effects.

## 4 Discussion

The present study examined the interplay between complex post-traumatic stress disorder (CPTSD), intimate partner violence (IPV), metacognitive beliefs about desire thinking, and perceived social support in explaining craving among women with methamphetamine use disorder. Overall, the findings provided strong support for a trauma-informed and metacognitive conceptualization of craving, highlighting both direct and indirect pathways through which trauma-related experiences contribute to craving severity.

### 4.1 CPTSD, IPV, and craving

Consistent with prior research, both CPTSD and IPV showed significant direct associations with craving. These findings support theoretical models that conceptualize trauma-related psychopathology and ongoing interpersonal stress as tonic vulnerability factors that heighten baseline susceptibility to craving (Sinha, 2013; Witkiewitz et al., 2014). CPTSD is characterized by pervasive affect dysregulation, negative self-concept, and chronic relational disturbances, all of which may compromise adaptive coping strategies and increase reliance on substances as maladaptive means of emotion regulation. IPV, as a chronic and proximal interpersonal stressor, may further exacerbate these processes by perpetuating fear, helplessness, and interpersonal insecurity.

Importantly, the present findings empirically support the conceptualization of IPV as both an etiological contributor to CPTSD and an independent predictor of craving. This distinction addresses a key theoretical concern by demonstrating that IPV is not merely subsumed under trauma-related symptomatology but exerts its own unique influence on craving severity. Examining CPTSD and IPV simultaneously within a single model therefore provides a more nuanced understanding of trauma-related risk processes among women with substance use disorders.

### 4.2 Mediating role of metacognitive beliefs about desire thinking

A central contribution of the present study lies in demonstrating that positive and negative metacognitive beliefs about desire thinking significantly mediated the relationships between both CPTSD and IPV with craving. These findings are highly consistent with metacognitive models of addictive behaviors (Spada et al., 2015) and align closely with recent systematic reviews and meta-analyses documenting robust associations between maladaptive metacognitive beliefs, desire thinking, and craving (Akbari et al., 2026; Mansueto et al., 2019).

Trauma-related symptoms may heighten the salience of internal cognitive and emotional experiences, fostering beliefs that engaging in desire thinking is both useful for coping and simultaneously uncontrollable or harmful (Myers & Wells, 2015, Spada et al., 2015). This combination of positive and negative metacognitions may paradoxically intensify craving by promoting repetitive cognitive elaboration while increasing distress related to perceived loss of cognitive control (Caselli and Spada, 2015, Mansueto et al., 2019). The absence of a significant mediating effect for the 'need to control thoughts' dimension suggests that not all metacognitive components contribute equally to craving among individuals in this population. Instead, beliefs concerning the utility and danger of desire thinking appear to play a particularly salient role among trauma-exposed women.

### 4.3 Role of perceived social support

Contrary to expectations, perceived social support did not significantly mediate the relationships between CPTSD, IPV, and craving, despite showing negative bivariate associations with both trauma symptoms and craving. This finding suggests that, in contexts characterized by chronic interpersonal trauma, proximal cognitive and metacognitive processes may outweigh broader interpersonal resources in shaping craving responses. Although social support is widely conceptualized as a protective factor that buffers the impact of stress on psychological outcomes (Cohen & Wills, 1985), some studies have shown its association with craving through mechanisms such as irrational beliefs (Bo et al., 2007). However, its effectiveness may be attenuated in populations exposed to ongoing interpersonal violence. For women experiencing IPV, social relationships may be characterized by ambivalence, reduced trust, or even coercive control, thereby limiting the accessibility and perceived reliability of support (Cohen and Wills, 1985, Dworkin et al., 2019, El-Bassel et al., 2000, El-Bassel et al., 2011, Thoits, 2011). Furthermore, trauma-related disturbances in self-organization (Favaretto et al., 2022), such as negative self-concept and interpersonal difficulties, may impair the ability to seek, perceive, or benefit from available support (Cloitre et al., 2018). These factors may help explain why perceived social support did not emerge as a significant mediator in the present model, despite its general relevance in the stress–coping literature.

### 4.4 Clinical implications

The findings have important clinical implications. First, they underscore the necessity of trauma-informed approaches in the treatment of methamphetamine use disorder among women, particularly interventions that address both CPTSD symptoms and ongoing interpersonal violence. Additionally, being engaged in work or meaningful activity can improve stress, craving, and mood for people with substance use disorder (Bertz et al., 2022). Prior research highlights that unaddressed trauma symptoms in substance abuse treatment are associated with poorer outcomes and higher relapse rates (Cosden et al., 2015; Froelich et al., 2022, Ibañez et al., 2022). Second, the results highlight metacognitive beliefs about desire thinking as modifiable treatment targets. Metacognitive therapy approaches, which specifically aim to modify maladaptive beliefs about thinking processes, have shown promise in reducing craving and addictive behaviors (Spada et al., 2015, Caselli and Spada, 2015). Integrating such techniques into existing treatment protocols may help individuals disengage from repetitive desire-related thinking patterns and reduce craving intensity. Clinically, these findings support the integration of trauma-focused and metacognitive interventions to directly target craving processes among women with substance use disorders, potentially enhancing treatment outcomes and reducing relapse vulnerability.

### 4.5 Limitations and future Directions

Several limitations of the present study should be acknowledged. First, the cross-sectional design precludes causal inferences regarding the observed relationships among trauma-related variables, metacognitive beliefs, and craving. Second, reliance on self-report measures may have introduced reporting and recall biases. Third, diagnostic assessments were based on clinical records and routine psychiatric evaluations rather than structured diagnostic interviews, which may have limited diagnostic precision. Additionally, the use of convenience sampling from treatment centers restricts the generalizability of the findings to broader populations of women with substance use disorders.

Future longitudinal and experimental studies are warranted to clarify causal pathways and to determine whether changes in metacognitive beliefs about desire thinking mediate treatment-related reductions in craving over time. Moreover, cultural factors related to gender roles and substance use stigma in Iran may have influenced the observed associations and should be explicitly examined in future cross-cultural research. Nevertheless, the proposed model is theory-driven and consistent with established metacognitive and trauma frameworks, lending conceptual robustness to the present findings despite these limitations.

## 5 Conclusion

The present study advances the understanding of craving among women with methamphetamine use disorder by integrating trauma-related and metacognitive perspectives within a single explanatory model. The findings demonstrate that both complex post-traumatic stress disorder and intimate partner violence are directly associated with craving and appear to indirectly influence craving through maladaptive metacognitive beliefs about desire thinking. In contrast, perceived social support did not exert a significant mediating effect, suggesting that proximal cognitive and metacognitive processes may play a more central role than broader interpersonal resources in trauma-exposed populations.

By empirically disentangling the roles of CPTSD and IPV, the present study underscores the importance of considering both cumulative trauma symptomatology and ongoing interpersonal violence when examining craving mechanisms among women. Moreover, the identification of metacognitive beliefs as key mediators highlights desire thinking as a clinically meaningful and modifiable process linking trauma exposure to craving severity.

Overall, the results support the integration of trauma-informed and metacognitive approaches in the treatment of methamphetamine use disorder among women. Targeting maladaptive metacognitive beliefs about craving-related cognitions may enhance treatment effectiveness and contribute to more personalized, mechanism-focused interventions for trauma-exposed individuals.

Role of funding Sources

This research did not receive any specific grant from funding agencies in the public, commercial, or not-for-profit sectors.

Informed Consent

Informed consent was obtained from all participants before they participated in the study.

Human and Animal Rights

This study, involving human participants, was conducted in accordance with the principles outlined in the Declaration of Helsinki.

## CRediT authorship contribution statement

**Roya Forootan:** Writing – review & editing, Writing – original draft, Software, Project administration, Investigation, Formal analysis, Data curation, Conceptualization. **Shahram Mohammadkhani:** Writing – review & editing, Writing – original draft, Validation, Supervision, Methodology, Investigation, Conceptualization. **Mehdi Akbari:** Writing – original draft, Writing – review & editing, Supervision, Methodology, Conceptualization. **Mark D. Griffiths:** Writing – review & editing, Validation, Methodology.

## Declaration of competing interest

The authors declare that they have no known competing financial interests or personal relationships that could have appeared to influence the work reported in this paper.

## Data Availability

Data are available upon reasonable request to the corresponding author.
